# Crystal structure of (*tert*-butyl­carbamo­yl)(4-chloro-2-oxo-2*H*-chromen-3-yl)methyl acetate

**DOI:** 10.1107/S2056989015021982

**Published:** 2015-11-28

**Authors:** Tetsuji Moriguchi, Venkataprasad Jalli, Suvratha Krishnamurthy, Akihiko Tsuge, Kenji Yoja

**Affiliations:** a Department of Applied Chemistry, Graduate School of Engineering, Kyushu Institute of Technology, 1-1 Sensui-cho, Tobata-ku, Kitakyushu 804-8550, Japan; b Japan Bruker AXS K.K.3-9, Moriya-cho Kanagawaku Yokohama 221-0022, Japan

**Keywords:** crystal structure, coumarin derivative, hydrogen bonding, π–π coumarin-ring inter­actions

## Abstract

In the title compound, C_17_H_18_ClNO_5_, which was synthesized by reacting 4-chloro-3-formyl­coumarin, acetic acid and *tert*-butyl isocyanide, the acetamido side chain is convoluted with ring-to-side chain C—C—C—C, C—C—C—N and C—C—N—C torsion angles of −123.30 (14), −135.73 (12) and 176.10 (12)°, respectively. In the crystal, N—H⋯O and weak C—H⋯O hydrogen bonds are present, which together with π–π coumarin-ring inter­actions [ring centroid separations = 3.4582 (8) and 3.6421 (9) Å], give rise to a layered structure lying parallel to (001).

## Related literature   

For applications of coumarin derivatives, see: Luo *et al.* (2012 ▸); Medina-Franco *et al.* (2011 ▸); Sun *et al.* (2013 ▸); Zen *et al.* (2014 ▸).
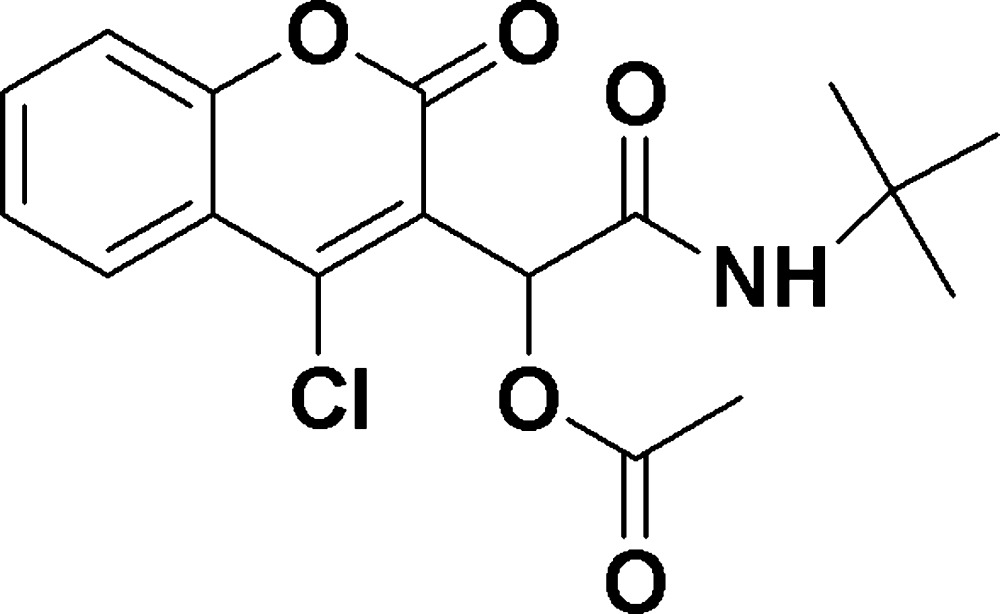


## Experimental   

### Crystal data  

C_17_H_18_ClNO_5_
*M* *_r_* = 351.77
Trigonal, 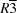

*a* = 29.831 (2) Å
*c* = 9.7983 (8) Å
*V* = 7551.2 (14) Å^3^
*Z* = 18
Mo *K*α radiation
μ = 0.25 mm^−1^
*T* = 90 K
0.50 × 0.45 × 0.45 mm

### Data collection  

Bruker APEXII diffractometer
Absorption correction: multi-scan (*SADABS*; Bruker, 2009 ▸) *T*_min_ = 0.746, *T*_max_ = 0.892
24469 measured reflections
2975 independent reflections
2742 reflections with *I* > 2σ(*I*)
*R*_int_ = 0.026

### Refinement  

*R*[*F* ^2^ > 2σ(*F* ^2^)] = 0.027
*wR*(*F* ^2^) = 0.094
*S* = 1.12
2975 reflections
221 parameters
H-atom parameters constrained
Δρ_max_ = 0.41 e Å^−3^
Δρ_min_ = −0.31 e Å^−3^

### 

Data collection: *APEX2* (Bruker, 2009 ▸); cell refinement: *SAINT* (Bruker, 2009 ▸); data reduction: *SAINT*; program(s) used to solve structure: *SHELXS97* (Sheldrick, 2008 ▸); program(s) used to refine structure: *SHELXL97* (Sheldrick, 2008 ▸); molecular graphics: *Mercury* (Macrae *et al.*, 2008 ▸); software used to prepare material for publication: *SHELXL97*.

## Supplementary Material

Crystal structure: contains datablock(s) global, I. DOI: 10.1107/S2056989015021982/zs2353sup1.cif

Structure factors: contains datablock(s) I. DOI: 10.1107/S2056989015021982/zs2353Isup2.hkl

Supporting information file. DOI: 10.1107/S2056989015021982/zs2353Isup3.pdf

Supporting information file. DOI: 10.1107/S2056989015021982/zs2353Isup4.cml

**Fig. 1.**
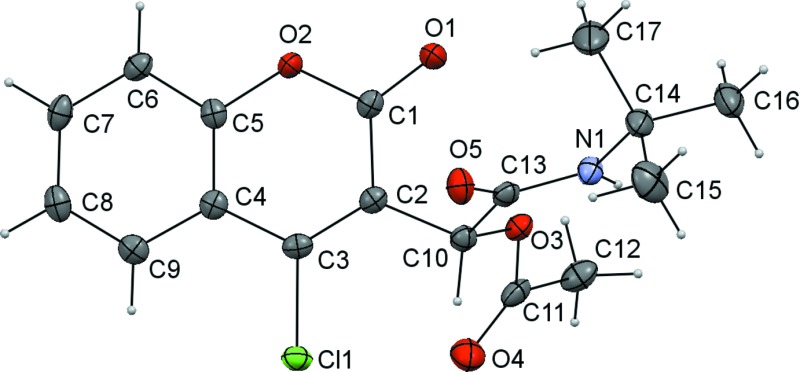
Molecular configuration and atom-numbering scheme for the title compound with displacement ellipsoids drawn at the 50% probability level.

**Fig. 2.**
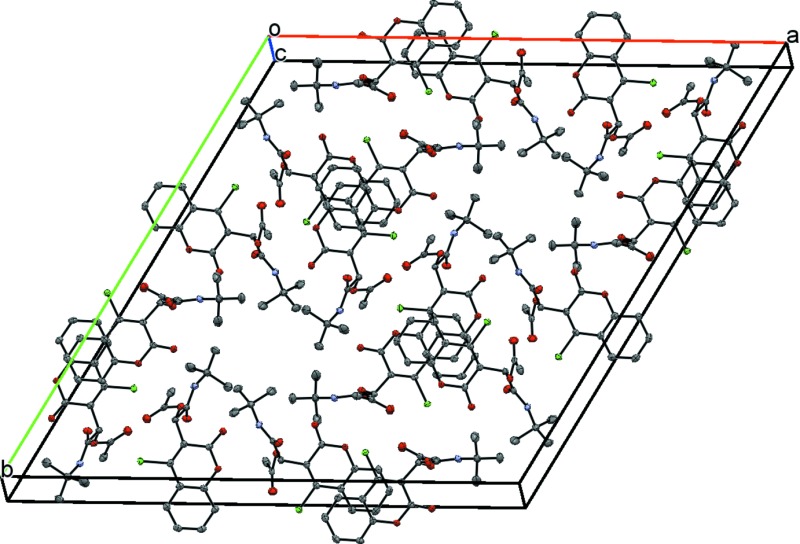
Crystal packing diagram of the title compound, viewed along the c axis, with hydrogen atoms omitted for clarity.

. DOI: 10.1107/S2056989015021982/zs2353fig1.tif
Mol­ecular configuration and atom-numbering scheme for the title compound with displacement ellipsoids drawn at the 50% probability level.

c . DOI: 10.1107/S2056989015021982/zs2353fig2.tif
Crystal packing diagram of the title compound, viewed along the *c* axis, with hydrogen atoms omitted for clarity.

**Fig. 3.**
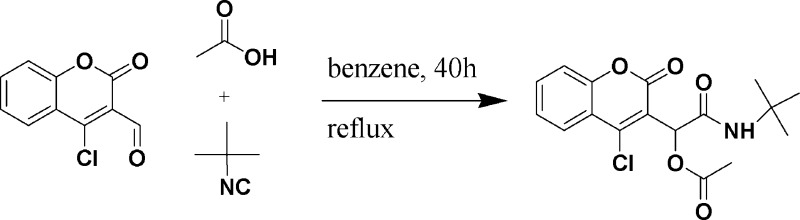
Reaction scheme for the synthesis of the title compound.

. DOI: 10.1107/S2056989015021982/zs2353fig3.tif
Reaction scheme for the synthesis of the title compound.

CCDC reference: 1437533

Additional supporting information:  crystallographic information; 3D view; checkCIF report

## Figures and Tables

**Table 1:** Hydrogen-bond geometry (Å, °)

*D*—H⋯*A*	*D*—H	H⋯*A*	*D*⋯*A*	*D*—H⋯*A*
N1—H1⋯O1^i^	0.86	2.33	3.0787 (16)	145
C16—H16*A*⋯O1^i^	0.96	2.56	3.287 (2)	133

Symmetry code: (i) 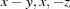.

## supplementary crystallographic information

### S1. Comment

Coumarin and its derivatives have gained significant importance due to their applications in various fields. 3-acetamido coumain derivatives isolated from plants used as DNA methyltransferase inhibitors for the development of cancer drugs (Medina-Franco *et al.*, 2011). 3-acetamido coumarin derivatives were also used as protein tyrosine phosphatase 1B (PTP 1B) inhibitors to develop effective drugs for diabetes and obesity (Sun *et al.*, 2013). Some of the coumarin derivatives were used as fluorescent sensors (Zen *et al.*, 2014). Natural coumarin derivatives isolated from plants such as microminutin, micromelin, psoralen and 8-methoxypsoralen have important properties in medicinal chemistry and bio-photochemistry (Luo *et al.* 2012). Thus, the elucidation of the crystal structures of coumarin derivatives has attracted much attention. Here,we report the crystal structure of the racemic title compound, C_17_H_18_ClNO_5_, which was synthesized by reacting 4-chloro-3-formyl coumarin, acetic acid and *tert*-butyl isocyanide in a one-pot reaction (Fig. 3).

In this compound (Fig. 1), the acetamido side chain is convoluted, with ring to side chain torsion angles C3—C2—C10—C13, C2—C10—C13—N1 and C10—C13—N1—C14 of -123.30 (14), -135.73 (12) and 176.10 (12)°, respectively. A number of intramolecular C—H···O, C—H···Cl and a N—H···O interactions are present. In the crystal, intermolecular N1—H···O1^i^ and weak C16—H···O1^i^ hydrogen bonds are present (Table 1) [for symmetry code (i), *x* - *y*, *x*, -*z*]. These, together with π–π coumarin ring interactions [ring centroid separations 3.4582 (8) and 3.6421 (9) Å], give a two-dimensional layered structure lying parallel to (001) (Fig. 2). The structure also has 34 Å^3^ solvent accessible voids.

### S2. Experimental

The title compound was synthesized as follows. A solution of 4-chloro-3-formyl coumarin (1 mmol), acetic acid (1 mmol) and *t*-butyl isocyanide (1 mmol) in 10 ml of benzene were refluxed at 80 ^0^C for 40h. The volatiles were removed under reduced pressure. The crude reaction mixture was subjected to column chromatography using an EtOAc/hexane mobile phase. The compound was isolated as a white colored solid with 70% yield. Single crystals of the title compound (m.p. 195–197 °C) suitable for X-ray analysis were obtained by slow room temperature evaporation of a dichloromethane solution. The molecule was crystallized in racemic form. Analysis: IR; ν_max_(KBr) 3144, 1735, 1680 cm^-1^; *δ*_H_ (500 MHz CDCl_3_) 7.97 (1 H, *J*=1.3 Hz, dd), 7.80 (1 H, m), 7.49-7.53 (2 H, m), 7.19 (1 H, s), 6.28 (1 H, s), 2.13 (3 H, s), 1.28 (9 H, s); *δ*_C_ (125 MHz, CDCl_3_) 168, 165, 158, 152, 150, 133, 126, 125, 122, 118, 116, 70, 52, 28, 20; LCMS: MH^+^, 350.

### S3. Refinement

All hydrogen atoms on aromatic C atoms and the N atom were placed in calculated positions and refined using a riding model, with C—H = 0.93–0.96 Å and N—H = 0.86 Å and with *U*_iso_(H) = 1.2*U*_eq_(aromatic C and N) or 1.5 *U*_eq_(methyl C). One reflection was considered to be affected by the beamstop.

### Crystal data

**Table:**

C_17_H_18_ClNO_5_	*D*_x_ = 1.392 Mg m^−^^3^
*M**_r_* = 351.77	Mo *K*α radiation, λ = 0.71073 Å
Trigonal, *R*3	Cell parameters from 24469 reflections
*a* = 29.831 (2) Å	θ = 1.4–25.0°
*c* = 9.7983 (8) Å	µ = 0.25 mm^−^^1^
*V* = 7551.2 (14) Å^3^	*T* = 90 K
*Z* = 18	Prism, colorless
*F*(000) = 3312	0.50 × 0.45 × 0.45 mm

### Data collection

**Table:**

Bruker APEXII diffractometer	2975 independent reflections
Radiation source: fine focus sealed tube	2742 reflections with *I* > 2σ(*I*)
Graphite monochromator	*R*_int_ = 0.026
Detector resolution: 8.333 pixels mm^-1^	θ_max_ = 25.0°, θ_min_ = 1.4°
ω scans	*h* = −35→35
Absorption correction: multi-scan (*SADABS*; Bruker, 2009)	*k* = −35→35
*T*_min_ = 0.746, *T*_max_ = 0.892	*l* = −11→11
24469 measured reflections	

### Refinement

**Table:**

Refinement on *F*^2^	Primary atom site location: structure-invariant direct methods
Least-squares matrix: full	Secondary atom site location: difference Fourier map
*R*[*F*^2^ > 2σ(*F*^2^)] = 0.027	Hydrogen site location: inferred from neighbouring sites
*wR*(*F*^2^) = 0.094	H-atom parameters constrained
*S* = 1.12	*w* = 1/[σ^2^(*F*_o_^2^) + (0.0575*P*)^2^ + 6.6693*P*] where *P* = (*F*_o_^2^ + 2*F*_c_^2^)/3
2975 reflections	(Δ/σ)_max_ = 0.001
221 parameters	Δρ_max_ = 0.41 e Å^−^^3^
0 restraints	Δρ_min_ = −0.31 e Å^−^^3^

### Special details

**Table:**

Geometry. All e.s.d.'s (except the e.s.d. in the dihedral angle between two l.s. planes) are estimated using the full covariance matrix. The cell e.s.d.'s are taken into account individually in the estimation of e.s.d.'s in distances, angles and torsion angles; correlations between e.s.d.'s in cell parameters are only used when they are defined by crystal symmetry. An approximate (isotropic) treatment of cell e.s.d.'s is used for estimating e.s.d.'s involving l.s. planes.
Refinement. Refinement of *F*^2^ against ALL reflections. The weighted *R*-factor *wR* and goodness of fit *S* are based on *F*^2^, conventional *R*-factors *R* are based on *F*, with *F* set to zero for negative *F*^2^. The threshold expression of *F*^2^ > σ(*F*^2^) is used only for calculating *R*-factors(gt) *etc*. and is not relevant to the choice of reflections for refinement. *R*-factors based on *F*^2^ are statistically about twice as large as those based on *F*, and *R*- factors based on ALL data will be even larger.

### Fractional atomic coordinates and isotropic or equivalent isotropic displacement parameters (Å^2^)

**Table:**

	*x*	*y*	*z*	*U*_iso_*/*U*_eq_	
C1	0.21876 (5)	−0.00044 (5)	0.08087 (13)	0.0160 (3)	
C2	0.26499 (5)	0.05104 (5)	0.07629 (13)	0.0157 (3)	
C3	0.31212 (5)	0.05536 (5)	0.08748 (13)	0.0155 (3)	
C4	0.31867 (5)	0.01052 (5)	0.09456 (13)	0.0158 (3)	
C5	0.27379 (5)	−0.03793 (5)	0.09425 (13)	0.0160 (3)	
C6	0.27504 (6)	−0.08370 (5)	0.09608 (14)	0.0188 (3)	
H6	0.2446	−0.1155	0.0958	0.023*	
C7	0.32278 (6)	−0.08086 (5)	0.09835 (14)	0.0208 (3)	
H7	0.3244	−0.1112	0.0992	0.025*	
C8	0.36839 (6)	−0.03316 (6)	0.09933 (14)	0.0206 (3)	
H8	0.4002	−0.0318	0.1015	0.025*	
C9	0.36650 (5)	0.01214 (5)	0.09716 (14)	0.0180 (3)	
H9	0.3971	0.0439	0.0974	0.022*	
C10	0.25496 (5)	0.09571 (5)	0.06509 (14)	0.0168 (3)	
H10	0.2879	0.1281	0.0746	0.02*	
C11	0.26696 (6)	0.12189 (5)	−0.16856 (15)	0.0206 (3)	
C12	0.23933 (6)	0.12088 (6)	−0.29691 (15)	0.0268 (3)	
H12A	0.2642	0.139	−0.3676	0.04*	
H12B	0.2191	0.1373	−0.281	0.04*	
H12C	0.2169	0.0856	−0.3244	0.04*	
C13	0.21854 (5)	0.09265 (5)	0.17996 (14)	0.0168 (3)	
C14	0.13982 (5)	0.09650 (6)	0.24241 (15)	0.0217 (3)	
C15	0.16453 (7)	0.13872 (7)	0.35092 (18)	0.0346 (4)	
H15A	0.1901	0.1347	0.4003	0.052*	
H15B	0.1384	0.136	0.4129	0.052*	
H15C	0.1807	0.1721	0.3078	0.052*	
C16	0.10059 (6)	0.10327 (7)	0.15898 (18)	0.0307 (4)	
H16A	0.1175	0.1366	0.1155	0.046*	
H16B	0.0738	0.1008	0.218	0.046*	
H16C	0.0857	0.0767	0.0907	0.046*	
C17	0.11322 (7)	0.04277 (7)	0.3076 (2)	0.0368 (4)	
H17A	0.1003	0.0169	0.2373	0.055*	
H17B	0.0849	0.0387	0.3633	0.055*	
H17C	0.1377	0.039	0.3631	0.055*	
Cl1	0.367571 (12)	0.115323 (12)	0.09482 (3)	0.01946 (13)	
N1	0.18014 (4)	0.10112 (4)	0.14609 (12)	0.0179 (3)	
H1	0.1787	0.1098	0.0632	0.021*	
O1	0.17497 (4)	−0.00851 (4)	0.07831 (10)	0.0197 (2)	
O2	0.22535 (4)	−0.04259 (3)	0.09066 (10)	0.0168 (2)	
O3	0.23274 (4)	0.09548 (4)	−0.06642 (9)	0.0183 (2)	
O4	0.31297 (4)	0.14258 (4)	−0.15359 (11)	0.0286 (3)	
O5	0.22830 (4)	0.08419 (4)	0.29548 (10)	0.0231 (2)	

### Atomic displacement parameters (Å^2^)

**Table:**

	*U*^11^	*U*^22^	*U*^33^	*U*^12^	*U*^13^	*U*^23^
C1	0.0201 (7)	0.0159 (7)	0.0134 (6)	0.0101 (6)	−0.0014 (5)	−0.0015 (5)
C2	0.0187 (7)	0.0151 (6)	0.0130 (6)	0.0083 (5)	0.0001 (5)	−0.0016 (5)
C3	0.0177 (7)	0.0143 (6)	0.0114 (6)	0.0056 (5)	0.0001 (5)	−0.0021 (5)
C4	0.0200 (7)	0.0183 (7)	0.0099 (7)	0.0102 (6)	−0.0009 (5)	−0.0018 (5)
C5	0.0181 (7)	0.0206 (7)	0.0116 (6)	0.0114 (6)	−0.0006 (5)	−0.0014 (5)
C6	0.0239 (7)	0.0165 (7)	0.0159 (7)	0.0102 (6)	−0.0007 (5)	−0.0004 (5)
C7	0.0307 (8)	0.0228 (7)	0.0163 (7)	0.0188 (6)	−0.0025 (6)	−0.0023 (5)
C8	0.0233 (7)	0.0304 (8)	0.0147 (7)	0.0184 (6)	−0.0024 (5)	−0.0032 (6)
C9	0.0190 (7)	0.0211 (7)	0.0130 (7)	0.0094 (6)	−0.0017 (5)	−0.0031 (5)
C10	0.0179 (7)	0.0148 (6)	0.0174 (7)	0.0081 (5)	−0.0018 (5)	−0.0018 (5)
C11	0.0276 (8)	0.0137 (6)	0.0229 (7)	0.0120 (6)	0.0069 (6)	0.0028 (5)
C12	0.0358 (9)	0.0211 (7)	0.0211 (7)	0.0122 (7)	0.0023 (6)	0.0034 (6)
C13	0.0186 (7)	0.0116 (6)	0.0195 (7)	0.0069 (5)	−0.0002 (5)	−0.0019 (5)
C14	0.0190 (7)	0.0223 (7)	0.0256 (8)	0.0117 (6)	0.0062 (6)	0.0052 (6)
C15	0.0316 (9)	0.0434 (10)	0.0342 (9)	0.0227 (8)	0.0053 (7)	−0.0095 (7)
C16	0.0223 (8)	0.0373 (9)	0.0371 (9)	0.0184 (7)	0.0061 (7)	0.0098 (7)
C17	0.0294 (9)	0.0349 (9)	0.0464 (10)	0.0163 (8)	0.0151 (8)	0.0199 (8)
Cl1	0.01575 (19)	0.01499 (19)	0.0235 (2)	0.00460 (13)	0.00001 (12)	−0.00316 (12)
N1	0.0199 (6)	0.0183 (6)	0.0176 (6)	0.0112 (5)	0.0021 (4)	0.0023 (4)
O1	0.0155 (5)	0.0169 (5)	0.0262 (5)	0.0077 (4)	−0.0018 (4)	−0.0009 (4)
O2	0.0161 (5)	0.0133 (4)	0.0215 (5)	0.0077 (4)	−0.0002 (4)	−0.0008 (4)
O3	0.0212 (5)	0.0165 (5)	0.0171 (5)	0.0094 (4)	0.0007 (4)	0.0023 (4)
O4	0.0242 (6)	0.0313 (6)	0.0299 (6)	0.0137 (5)	0.0078 (4)	0.0079 (5)
O5	0.0262 (5)	0.0297 (6)	0.0193 (5)	0.0184 (5)	−0.0013 (4)	−0.0007 (4)

### Geometric parameters (Å, º)

**Table:**

C1—O1	1.2045 (16)	C11—O3	1.3638 (17)
C1—O2	1.3695 (16)	C11—C12	1.496 (2)
C1—C2	1.4644 (18)	C12—H12A	0.96
C2—C3	1.3507 (19)	C12—H12B	0.96
C2—C10	1.5085 (18)	C12—H12C	0.96
C3—C4	1.4469 (18)	C13—O5	1.2260 (17)
C3—Cl1	1.7269 (13)	C13—N1	1.3325 (18)
C4—C5	1.3952 (19)	C14—N1	1.4802 (17)
C4—C9	1.4033 (19)	C14—C16	1.521 (2)
C5—O2	1.3814 (16)	C14—C15	1.527 (2)
C5—C6	1.3843 (19)	C14—C17	1.528 (2)
C6—C7	1.384 (2)	C15—H15A	0.96
C6—H6	0.93	C15—H15B	0.96
C7—C8	1.393 (2)	C15—H15C	0.96
C7—H7	0.93	C16—H16A	0.96
C8—C9	1.380 (2)	C16—H16B	0.96
C8—H8	0.93	C16—H16C	0.96
C9—H9	0.93	C17—H17A	0.96
C10—O3	1.4475 (16)	C17—H17B	0.96
C10—C13	1.5352 (19)	C17—H17C	0.96
C10—H10	0.98	N1—H1	0.86
C11—O4	1.1998 (18)		
			
O1—C1—O2	117.20 (12)	C11—C12—H12B	109.5
O1—C1—C2	124.58 (12)	H12A—C12—H12B	109.5
O2—C1—C2	118.21 (11)	C11—C12—H12C	109.5
C3—C2—C1	119.15 (12)	H12A—C12—H12C	109.5
C3—C2—C10	125.34 (12)	H12B—C12—H12C	109.5
C1—C2—C10	115.46 (11)	O5—C13—N1	125.74 (13)
C2—C3—C4	122.08 (12)	O5—C13—C10	117.08 (12)
C2—C3—Cl1	120.95 (10)	N1—C13—C10	117.14 (12)
C4—C3—Cl1	116.97 (10)	N1—C14—C16	106.75 (12)
C5—C4—C9	117.92 (12)	N1—C14—C15	109.41 (12)
C5—C4—C3	117.02 (12)	C16—C14—C15	110.54 (13)
C9—C4—C3	125.02 (12)	N1—C14—C17	109.62 (12)
O2—C5—C6	116.35 (12)	C16—C14—C17	109.48 (13)
O2—C5—C4	121.20 (11)	C15—C14—C17	110.95 (14)
C6—C5—C4	122.45 (12)	C14—C15—H15A	109.5
C7—C6—C5	118.30 (13)	C14—C15—H15B	109.5
C7—C6—H6	120.8	H15A—C15—H15B	109.5
C5—C6—H6	120.8	C14—C15—H15C	109.5
C6—C7—C8	120.80 (13)	H15A—C15—H15C	109.5
C6—C7—H7	119.6	H15B—C15—H15C	109.5
C8—C7—H7	119.6	C14—C16—H16A	109.5
C9—C8—C7	120.20 (13)	C14—C16—H16B	109.5
C9—C8—H8	119.9	H16A—C16—H16B	109.5
C7—C8—H8	119.9	C14—C16—H16C	109.5
C8—C9—C4	120.32 (13)	H16A—C16—H16C	109.5
C8—C9—H9	119.8	H16B—C16—H16C	109.5
C4—C9—H9	119.8	C14—C17—H17A	109.5
O3—C10—C2	110.68 (10)	C14—C17—H17B	109.5
O3—C10—C13	110.09 (11)	H17A—C17—H17B	109.5
C2—C10—C13	109.95 (11)	C14—C17—H17C	109.5
O3—C10—H10	108.7	H17A—C17—H17C	109.5
C2—C10—H10	108.7	H17B—C17—H17C	109.5
C13—C10—H10	108.7	C13—N1—C14	124.00 (12)
O4—C11—O3	122.73 (13)	C13—N1—H1	118.0
O4—C11—C12	126.18 (13)	C14—N1—H1	118.0
O3—C11—C12	111.08 (12)	C1—O2—C5	122.19 (10)
C11—C12—H12A	109.5	C11—O3—C10	116.22 (11)
			
O1—C1—C2—C3	176.34 (13)	C3—C4—C9—C8	−177.79 (12)
O2—C1—C2—C3	−2.70 (19)	C3—C2—C10—O3	114.86 (14)
O1—C1—C2—C10	−1.13 (19)	C1—C2—C10—O3	−67.85 (14)
O2—C1—C2—C10	179.83 (11)	C3—C2—C10—C13	−123.30 (14)
C1—C2—C3—C4	4.3 (2)	C1—C2—C10—C13	53.99 (15)
C10—C2—C3—C4	−178.51 (12)	O3—C10—C13—O5	168.64 (11)
C1—C2—C3—Cl1	−175.38 (9)	C2—C10—C13—O5	46.45 (16)
C10—C2—C3—Cl1	1.81 (19)	O3—C10—C13—N1	−13.54 (16)
C2—C3—C4—C5	−2.2 (2)	C2—C10—C13—N1	−135.73 (12)
Cl1—C3—C4—C5	177.46 (9)	O5—C13—N1—C14	−6.3 (2)
C2—C3—C4—C9	175.57 (13)	C10—C13—N1—C14	176.10 (12)
Cl1—C3—C4—C9	−4.74 (19)	C16—C14—N1—C13	−173.64 (13)
C9—C4—C5—O2	−179.48 (11)	C15—C14—N1—C13	66.72 (17)
C3—C4—C5—O2	−1.52 (19)	C17—C14—N1—C13	−55.16 (18)
C9—C4—C5—C6	−0.1 (2)	O1—C1—O2—C5	179.92 (11)
C3—C4—C5—C6	177.82 (12)	C2—C1—O2—C5	−0.97 (18)
O2—C5—C6—C7	179.39 (11)	C6—C5—O2—C1	−176.28 (11)
C4—C5—C6—C7	0.0 (2)	C4—C5—O2—C1	3.10 (19)
C5—C6—C7—C8	0.3 (2)	O4—C11—O3—C10	2.96 (18)
C6—C7—C8—C9	−0.4 (2)	C12—C11—O3—C10	−177.30 (11)
C7—C8—C9—C4	0.3 (2)	C2—C10—O3—C11	−89.05 (13)
C5—C4—C9—C8	0.0 (2)	C13—C10—O3—C11	149.19 (11)

### Hydrogen-bond geometry (Å, º)

**Table:**

*D*—H···*A*	*D*—H	H···*A*	*D*···*A*	*D*—H···*A*
N1—H1···O3	0.86	2.25	2.6627 (17)	109
N1—H1···O1^i^	0.86	2.33	3.0787 (16)	145
C9—H9···Cl1	0.93	2.68	3.0623 (15)	105
C10—H10···Cl1	0.98	2.60	3.1220 (17)	114
C15—H15*A*···O5	0.96	2.52	3.107 (3)	120
C16—H16*A*···O1^i^	0.96	2.56	3.287 (2)	133
C17—H17*C*···O5	0.96	2.43	3.014 (3)	119

Symmetry code: (i) *x*−*y*, *x*, −*z*.
