# Supplementary figures and images for: Crystal structure of (tert-butyl­carbamo­yl)(4-chloro-2-oxo-2H-chromen-3-yl)methyl acetate
Source: Acta Crystallogr E Crystallogr Commun. 2015 Nov 28;71(Pt 12):o1002. doi: 10.1107/S2056989015021982 (PMC4719945; doi:10.1107/S2056989015021982)

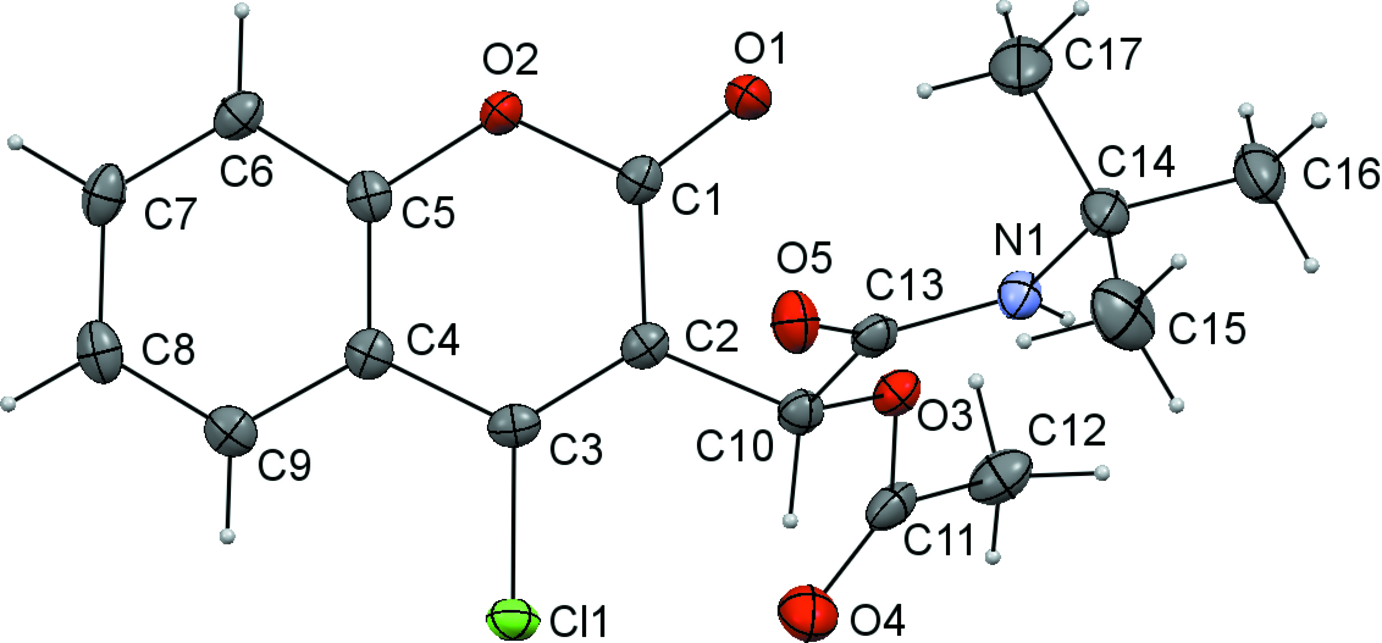

Supplement: Supplementary file 5 [file e-71-o1002-fig1.tif]

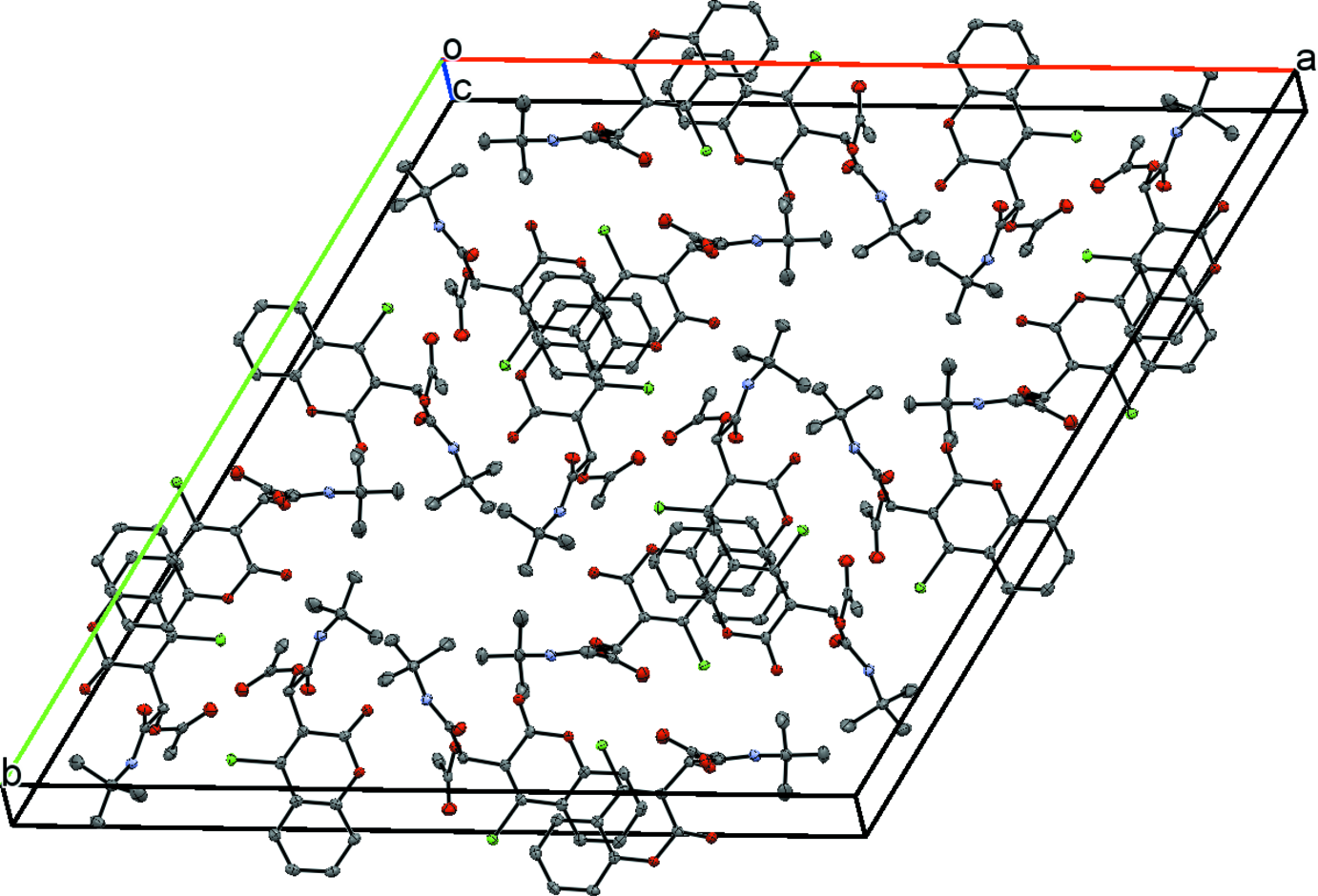

Supplement: Supplementary file 6 [file e-71-o1002-fig2.tif]
